# Relationships between Nutrient Heterogeneity, Root Growth, and Hormones: Evidence for Interspecific Variation

**DOI:** 10.3390/plants7010015

**Published:** 2018-02-28

**Authors:** Jia Dong, Robert H. Jones, Pu Mou

**Affiliations:** 1 State Key Laboratory of Earth Surface Processes and Resource Ecology and Ministry of Education Key Laboratory for Biodiversity Science and Engineering, Beijing Normal University, Beijing 100875, China; dongjia1@illinois.edu; 2 Department of Forestry and Environmental Conservation, Clemson University, Clemson, SC 29634, USA; provost@clemson.edu

**Keywords:** root growth, root hormones, root architecture, nitrogen treatment

## Abstract

(1) Background: Plant roots respond to nutrients through root architecture that is regulated by hormones. Strong inter-specific variation in root architecture has been well documented, but physiological mechanisms that may control the variation have not. (2) Methods: We examined correlations between root architecture and hormones to seek clues on mechanisms behind root foraging behavior. In the green house at Beijing Normal University, hydroponic culture experiments were used to examine the root responses of four species—*Callistephus chinensis*, *Solidago canadensis*, *Ailanthus altissima*, *Oryza sativa—*to two nitrogen types (NO_3_^−^ or NH_4_^+^), three nitrogen concentrations (low, medium, and high concentrations of 0.2, 1, and 18 mM, respectively) and two ways of nitrogen application (stable vs. variable). The plants were harvested after 36 days to measure root mass, 1st order root length, seminal root length for *O. sativa*, density of the 1st order laterals, seminal root number for *O. sativa*, the inter-node length of the 1st order laterals, and root hormone contents of indole-3-acetic acid, abscisic acid, and cytokinins (zeatin + zeatinriboside). (3) Results: Species differed significantly in their root architecture responses to nitrogen treatments. They also differed significantly in hormone responses to the nitrogen treatments. Additionally, the correlations between root architecture and hormone responses were quite variable across the species. Each hormone had highly species-specific relationships with root responses. (4) Conclusions: Our finding implies that a particular root foraging behavior is probably not controlled by the same biochemical pathway in all species.

## 1. Introduction

Soil resources are heterogeneously distributed in space and time at various scales, and with differences in concentration that individual plants sense and respond to [1,2]. Consequently, plants often develop asymmetric root systems or adjust resource uptake rates [3], both leading to what is known as root foraging plasticity [4,5].

Specifically, root foraging plasticity has been defined as phenotypical changes of roots under the influences of soil variation [6,7,8], and the changes have been classified as morphological, physiological, demographic, and mycorrhizal plasticity [9,10,11]. Through these types of plasticity, plants are able to efficiently acquire resources in heterogeneous resource environments [5,12]. Most studies of root foraging behavior have focused on morphological plasticity, i.e., the changes in root proliferation and architecture features in resource patchy soils [11,13].

Root architecture (RA) refers to the spatial configuration of the root systems, or the explicit geometric deployment of root axes applying to either an entire root system or a subset of the root system of an individual plant [14]. Root architecture is regulated by responses to environmental cues that trigger changes in hormones and downstream responses of cells and tissues [15,16].

It is well known that uptake of nitrogen, especially in its two common forms nitrate and ammonium, has a large impact on the configuration of RA [17,18,19]. Nitrate acts as an external signal and has been reported to directly stimulate primary and lateral root growth and affect RA in *Arabidopsis*. These root responses are triggered by the perception of the NO_3_^−^ ion, which takes place at the primary root tip [19,20,21]. Ammonium in high concentrations can stunt root growth, although the toxicity of ammonium is poorly understood. It has been established that similar concentrations of nitrate and ammonium can have vastly different effects on root growth and architecture. At high concentrations (10 mM), NH4^+^ more severely inhibited root growth in tomato as compared to similar concentrations of NO_3_^−^ [22], whereas at low concentration of 1 mM, the primary root grew larger with NH4^+^ than with NO_3_^−^ in maize [23]. 

Phytohormones regulate and influence root growth and architecture through diverse mechanisms [24,25]. Indole-3-acetic acid (IAA) influences the formation of primary and lateral roots [26,27]; the behavior of the quiescent center [28], root apical meristem [29], and root cap [30]; root vascular differentiation [24,31]; and, the growth of lateral roots [32]. Cytokinins (CKs) are synthesized in the root tip and promote cytokinesis, vascular cambium sensitivity, vascular differentiation, and root apical dominance [24]. Cytokinins (CKs) generally suppress root growth and development [33] and have antagonistic interactions with IAA in roots [34]. 

Hormones serve as the internal mediators between soil conditions and RA responses during root development [15,35,36]. A large body of work, often using *Arabidopsis* as a model, has shown that nitrate triggers many of the root responses [15,18,24,37,38,39,40,41]. A general framework has been proposed that links environmental nutrient conditions to hormonal regulation and down steam growth responses, including root morphology [37].

Abscisic acid (ABA) is produced in roots and is commonly believed to be involved in abiotic and biotic stress responses [42,43], but its role in regulating root growth is not fully understood [42,44]. ABA inhibited the emergence of lateral root (LR) primordia [45], but data from a study in *Arabidopsis* revealed that ABA signaling is necessary for auxin-mediated LR formation, indicating a coordination between ABA signaling and auxin [25,46]. In this study, we examined the relationships between and among patterns of nutrient supply, root hormones, and RA features in four plant species with various ecological strategies: *Callistephus chinensis* (Chinese aster), an annual/biannual herb, *Ailanthus altissima* (Tree of Heaven), a deciduous hardwood tree, *Oryza sativa* (rice-cultivar), a monocot gramineous species, and *Solidago canadensis* (Canadian goldenrod), a perennial herb. In a hydroponic experiment, we varied nitrogen types (NO_3_^−^ or NH4^+^), three nitrogen concentrations (low, medium, and high concentrations of 0.2, 1, and 18 mM, respectively) and two ways of nitrogen application (stable vs. variable: low, medium and high concentrations in the stable application, and shifting between low and high, and between medium and high in the variable application), and measured responses of RA features and three hormones (IAA, ABA, and CK) that regulate root growth and development in plants. While we were certain that interspecific variation would occur in RA responses to nutrient treatments, we were less certain that this would be true for hormone responses because much of the literature on this has been conducted using single-species experiments, usually with model species such as *Arabidopsis* or Maize (*Zea mays*). When considering that interspecific comparison has not been frequently done, we were particularly interested in exploring the correlations between hormone contents and RA behavior across species with very different phylogenies and ecological characteristics. Common patterns of correlation would suggest common physiological mechanisms controlling RA responses to soil heterogeneity. This study has a potential to expand findings beyond model species, such as *Arabidopsis* to other plants in plant communities.

## 2. Materials and Methods

### 2.1. Plant Material and Culture Conditions

The experiment was carried out in an environmentally controlled greenhouse at Beijing Normal University. The seeds of the four species came from different sources: *C. chinensis* was obtained from Xinnongfeng Inc., of China (No. 12 Zhongguancun S. Avenue, Beijing, China), *S. canadensis* was purchased from a commercial source (ERNST Conservation Seeds LP, Meadville, PA, USA), *A. altissima* was collected from the Beijing Normal University campus, and *O. sativa*-cultivar Zhonghua No. 11 was provided by the Institute of Genetics, Chinese Academy of Sciences, Beijing, China. The seeds were submerged in 1% (*v*/*v*) H_2_O_2_ solution for 30 min for surface sterilization, and rinsed three times with deionized water before sowing in pans that are filled with construction grade sand. Seedlings were transplanted into water tanks for nitrogen treatments when they grew to the following sizes (stages): six leaves present for *C. chinensis*, eight leaves for *S. canadensis*, 10 cm tall for *A. altissima*, and presence of second leaf stage for *O. sativa*. The dark colored plastic water tanks (Length × Width × Height = 46 × 30 × 14 cm) were filled with 10 liters of Hoagland nutrient solution at one of three concentrations of NO_3_^−^ or NH4^+^. The solution was aerated with pumps continuously. A polyvinyl chloride plate (40 × 25 cm) with 24 holes of 0.5 cm in diameter and 6 cm apart from each other was floated in each tank. The polyvinyl chloride plates were large enough to cover the water surface, with dark colored tanks together, substantially reduced the light of rooting waters to minimize possible effects on root growth. Twenty-four seedlings of a single species were transplanted to the plate with their roots pushed through the holes into the hydroponic solution. Plants were grown in a greenhouse with a 16 h light/8 h dark photoperiod. The air temperature ranged between 23–28 °C in the daytime and 15–20 °C at night. Relative humidity was maintained at 35 ± 5% in the daytime and 55 ± 5% at night. Light intensity at the top of the plant canopy was approximately 300 µmol m^−2^ s^−1^ (photosynthetic photon flux density (PPFD)).

Earlier studies have focused on root responses to stable nitrogen treatment. Here, we added another dimension to examine the effect of variable nitrogen concentration on root architecture. We employed three stable N concentrations of 0.2 mM, 1 mM, and 18 mM, and two types of variable N concentrations in this study.

### 2.2. Nitrogen Treatment

**Figure 1:**
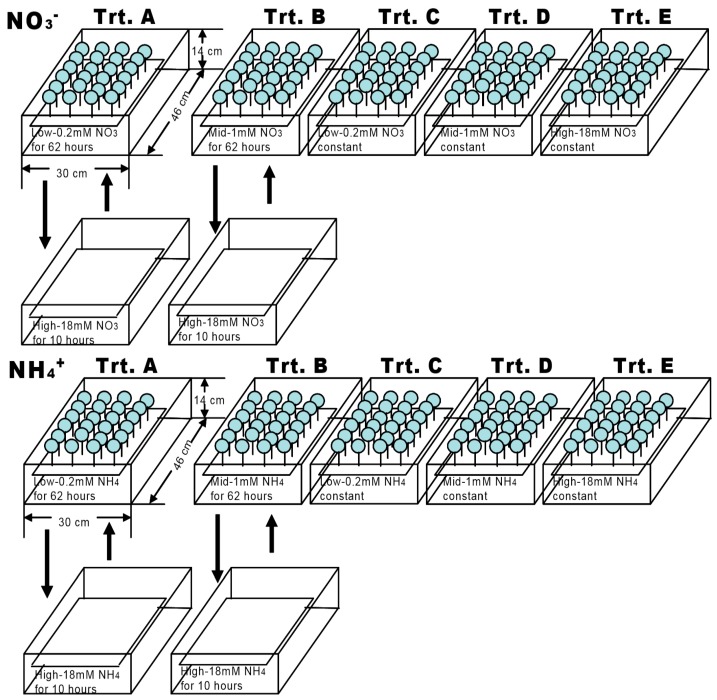
An illustration of five treatments under each N type (NO_3_^–^ and NH_4_^+^). Trt. A: variable N concentrations of low/high, Trt. B: variable N concentrations of mid/high, Trt. C: stable N concentration of 0.2 mM, Trt. D: stable N concentration of 1 mM, Trt. E: stable N concentration of 18 mM. Trt.: Treatment.

The nutrient treatments were applied (Figure 1) as the plants were transplanted. Three concentrations, low (0.2 mM), medium (1 mM), and high (18 mM) of NO_3_^−^ and NH4^+^ were used consistently throughout the experiment (stable treatment), or the plants were shifted between low and high or between medium and high on a 72 h cycle (62 h at the lower concentration followed by 10 h at the high concentration) (variable treatment). 

In total, we had ten treatments for each plant species and five treatments for NO_3_^−^ and NH4^+^ each, i.e., treatment (A) variable N concentrations of low/high (62 h at 0.2 mM followed by 10 h at 18 mM); (B) variable N concentrations of mid/high (62 h at 1 mM followed by 10 h at 18 mM); (C) stable N concentration of 0.2 mM; (D) stable N concentration of 1 mM; and, (E) stable N concentration of 18 mM (Table 1 and Figure 1). 

To maintain relatively stable N levels throughout the experiment, we completely refreshed the solutions every six days in accordance with protocols used in previous hydroponic studies [47,48]. Solutions in the tanks were refilled to 10 L every day to replace the loss through evapo-transpiration.

Modified Hoagland nutrient solutions with different NO_3_^−^ (using KNO_3_ and Ca(NO_3_)_2_) or NH_4_^+^ (using NH_4_Cl and (NH_4_)_2_SO_4_) concentrations were prepared for each treatment using de-ionized water (Table 2). In this study, all solutions were balanced. The micronutrients, ferric salt and macronutrients (K, P, Ca and Mg) were kept constant for all treatments and plants. Second, the NO_3_^−^ treatments (0.2 mM, 1 mM and 18 mM) were supplemented with CaCl_2_ and KCl to maintain Ca^2+^ and K^+^ at a 6 mM level. The pH of all treatment solutions was daily adjusted to pH 6.0 with 1 mol/L NaOH or HCl. The experiment continued for 36 days and the variable nitrogen concentration treatments were repeated 12 times.

### 2.3. Harvest

At the end of the experiment, we randomly selected five healthy individuals of each species in each treatment for harvest. The root system of each harvested plant was carefully picked from the solution with a pair of tweezers, and put into a large glass pan with 1 cm deep treatment solution. The root system was gently spread out on a white plastic sheet, and scanned with an EPSON root scanner (G780B, Seiko Epson Corporation, Tokyo, Japan). After scanning, 0.05 ± 0.005 g of fresh lateral roots were cut with scissors, quickly packed with an aluminum foil sheet, and were stored in a thermos with liquid nitrogen for later hormone extraction. The fresh weights of the shoots and the remaining roots were measured, and then their dry weights were determined (oven dried at 65 °C to consistent weights).

### 2.4. Root Architecture (RA) Measurement and Analysis

With the scanned pictures of the root systems, the length of the 1st order fine roots (1st ORL) was recorded, the inter-branch length of the 1st order laterals (IBLLR), and the density of the 1st order laterals (1st LRD) were measured from at least 10 randomly chosen 2nd order root axils. For the monocot *O. sativa*, which has a fibrous root system different from the other three species, we counted the number of seminal roots (SR #) and measured the total seminal root (SR) length from the scanned graph. All of the length measurements including the 1st LRD were conducted by using ArcGIS10.0 software (Esri, Redlands, CA, USA). 

### 2.5. Hormone Extraction and Purification

Indole-3-acetic acid (IAA), abscisic acid (ABA) and cytokinins (CK(Z+ZR)) were extracted from the frozen fine root samples and purified following previous studies with minor modification [49,50]. Each root sample was ground in an ice-cooled mortar in 1 mL 80% (*v*/*v*) methanol extraction medium containing 1 µmol butylatedhydroxytoluene as an antioxidant. The ground sample was incubated at 4 °C for 4 h and centrifuged at 4000 rpm for 15 min at 4 °C. The supernatant was passed through Chromosep C18 columns (C18 Sep-Park Cartridge, Waters Corporation, Millford, MA, USA) and prewashed with 10 mL 100% (*v*/*v*) and 5 mL 80% (*v*/*v*) methanol solutions, respectively. The hormone fractions eluted with 10 mL 100% (*v*/*v*) methanol and 10 mL ether from the columns were dried and dissolved in 0.5 mL phosphate buffer saline (PBS) containing 0.1% (*v*/*v*) Tween 20 and 0.1% (*w*/*v*) gelatin (pH 7.5), for analysis by Enzyme-Linked Immunosorbent Assay (ELISA).

### 2.6. Quantification of Hormones by ELISA

Indole-3-acetic acid (IAA), ABA, and CKs (Zeatin and ZeatinRiboside, CK(Z+ZR) hereafter) were extracted and purified following a previous study [51]. Mouse monoclonal antigens and antibodies for IAA, ABA, and CK(Z+ZR), and IgG-horseradish peroxidase used in ELISA were from the Phytohormone Institute, China Agricultural University [50]. Enzyme-Linked Immunosorbent Assay (ELISA) was performed on a 96-well micro-titration plate. Each well was coated with 100 µL buffer (1.5 g L^−1^ Na_2_CO_3_, 2.93 g L^−1^ NaHCO_3_, and 0.02 g L^−1^ NaN_3_, pH 9.6) containing 0.25 µg mL^−1^ of antigens. The coated plates were incubated at 37 °C for 4 h for ABA and CK(Z+ZR) analysis, and at 4 °C overnight for IAA. The plates were then kept at room temperature for 30 min and washed four times with PBS + Tween 20 (0.1% (*v*/*v*)) buffer (pH 7.4). Then, the wells of each plate were filled with 50 µL of either grain extracts or standards (0–2000 ng mL^−1^ dilution range) of IAA, ABA, and CK(Z+ZR). Next, 50 µL of 20 µg mL^−1^ of antibodies for IAA, ABA, or CK(Z+ZR), were added. Plates were incubated at 37 °C for 30 min and washed again, as described above. Each well then received 100 µL of 1.25 µg mL^−1^ IgG-horseradish peroxidase substrate and the plate was incubated at 37 °C for another 30 min. The plate was then rinsed five times with PBS + Tween 20 buffer, and 100 µL color-appearing solution containing 1.5 mg mL^−1^ ***o*-**phenylenediamine. Next, 0.008% (*v*/*v*) H_2_O_2_ was added to each well to catalyze the enzyme reaction. When the standard solutions developed a color gradient with the 0 ng mL^−1^ standard having the deepest color, the reaction was stopped by adding 50 µL of 2 mol L^−1^ H_2_SO_4_ to each well. Color development in each well was detected using an ELISA Reader (model EL310, Bio-TEK, Winooski, VT, USA) at optical density A490. Indole-3-acetic acid (IAA), ABA, and CK (Z+ZR) contents were calculated, as described previously [52].

### 2.7. Statistical Analysis

To examine the relationships among N variables, RA variables and hormone responses, we used Multivariate analysis of variance (MANOVA) and regression analyses. MANOVA was performed on each plant species to examine the effects of nitrogen variability (NO_3_^−^, NH_4_^+^, stable vs. variable, and varied concentrations) on RA variables (1st ORL, IBLLR, and 1st OLRD), and on hormone variables (IAA, ABA, and CK(Z+ZR)). MANOVA was chosen because root mass, 1st ORL, IBLLR, and 1st OLRD and concentrations of IAA, ABA, and CK(Z+ZR) in roots may be correlated. Prior to MANOVA, we determined if the data sets for root mass, 1st ORL, IBLLR, 1st OLRD (for *O. sativa*, total SR length, the SR#), IAA, ABA, and CK(Z+ZR) were homoscedastic, and if they were not, we conducted a log transformation. Protected ANOVA (under MANOVA) followed by multiple comparisons was used when necessary to explore specific differences among the root responses of specific N treatments [53,54].

Because we found that RA and hormone responses to NO_3_^−^ treatments differed from those under NH_4_^+^ treatments, we investigated the quantitative relationships between RA variables and hormones for each nutrient type separately. For each nutrient source, multivariate regression was used with each RA variable as the response variable and the hormones as concomitant variables, with no assumptions of cause and effect made. All of the statistical analyses were performed using SPSS 20.0 software (IBM^®^ SPSS^®^ Statistics (Armonk, NY, USA)).

## 3. Results

### 3.1. RA Responses to N Treatments

**Figure 2:**
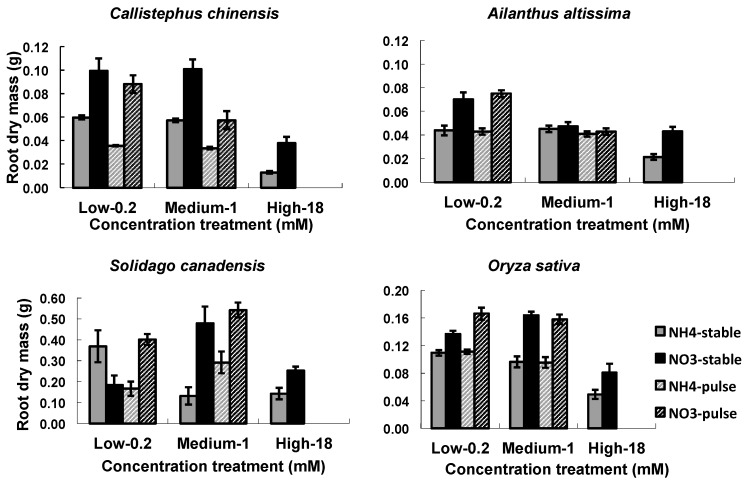
Nitrogen types, three nitrogen concentrations and two ways of nitrogen application on root dry mass. Nitrogen types refer to NO_3_^–^ vs. NH_4_^+^, three nitrogen concentrations refer to low, medium, and high concentrations of 0.2, 1, and 18 mM, respectively, two ways of nitrogen application refers to stable vs. variable. In each graph, four bars indicated responses of the root dry mass to the NO_3_^–^ (black), NH_4_^+^ (grey), variable N (grey striped), and stable (black striped) treatments. Each column of graphs represents one species; error bars are standard errors. Each bar represents five plants.

**Figure 3:**
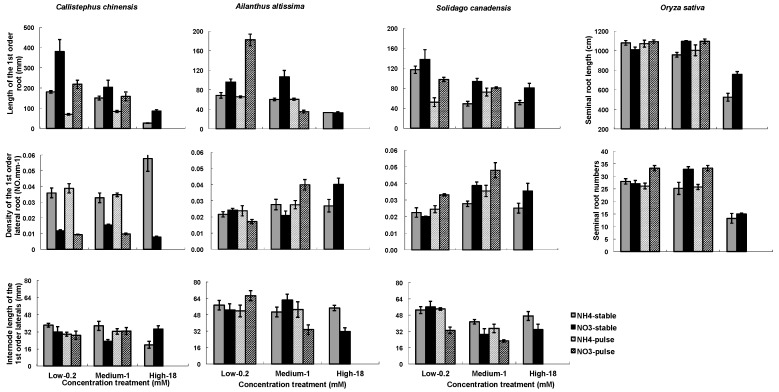
Nitrogen types, three nitrogen concentrations and two ways of nitrogen application on root architecture. Root architecture include of length of the 1st order root (1st ORL) or seminal root length for rice, the inter-node length of the 1st order laterals (IBLLR), density of the 1st order lateral roots (1st ORD). Each column of graphs represents one species; error bars are standard errors. Each bar represents five plants.

The two nitrogen types (NO_3_^−^ or NH_4_^+^) and the concentrations (low, medium, high) influenced RA in all four species, while the nitrogen application (stable or variable) treatments influenced RA in *C. chinensis* and in *S. canadensis* (Table 3). There were significant interactions among the three treatment factors (Table 3). In general, root mass and length (1st ORL) were greater in NO_3_^−^ treatments than in NH_4_^+^, and were greater in the low and medium than in the high concentration (Figure 2 and Figure 3). Densities of 1st order laterals (1st LRD) were highly variable across nitrogen types and concentrations with no consistent patterns across the species, and the inter-branch length of the 1st order laterals (IBLLR) varied relatively little in comparison with the other RA variables (Figure 3). When compared to stable treatments, variable treatments decreased the root dry mass and the length of 1st order root in *C. chinensis*, decreased the total root biomass in low/high ammonium treatment in *S. canadensis*, decreased length of 1st order roots in low/high treatment in *S. canadensis* and in medium/high NO_3_^−^ treatment in *A. attissima* but had no significant effect on the total root dry mass in *A. attissima* and *O. sativa* (Figure 2 and Figure 3).

### 3.2. Hormone Responses of Fine Roots to N Treatment

**Figure 4:**
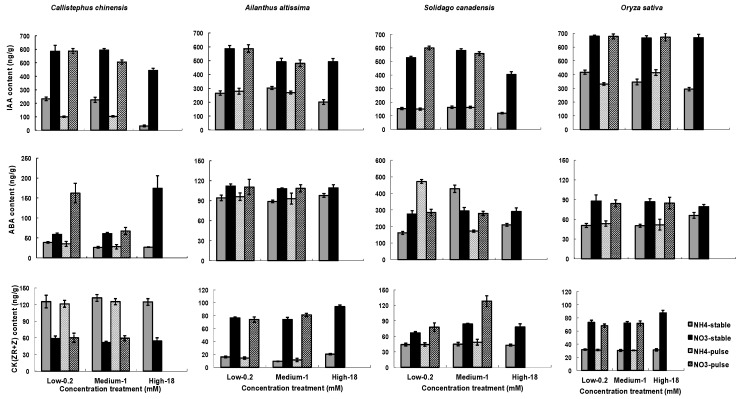
Influence of nitrogen types, three nitrogen concentrations and two ways of nitrogen application on hormones in roots. Hormones include of concentrations of IAA, ABA, and CK(ZR+R) in roots. Each column of graphs represents one species; error bars are standard errors. Each bar represents five plants.

Hormone contents were significantly influenced by treatments (Table 3). N type had a much stronger and more consistent effect across species than did nutrient concentration or temporal application of nutrients (Figure 4). All of the species demonstrated much higher IAA contents in the NO_3_^−^ than in the NH_4_^+^ treatments, and in general, root IAA content was lower at 18 mM NO_3_^−^ or NH_4_^+^ than at the other two tested nitrogen concentrations, with the exception of at 18 mM NO_3_^−^ or NH_4_^+^ in *Oryza sativa* (Figure 4). Root IAA content of *C. chinensis* decreased significantly in variable treatments than in stable treatments, but not of the other three species. Root ABA content tended to be greater in the NO_3_^−^ than in the NH_4_^+^treatment as well, except in *S. canadensis* where a considerable variability was observed (Figure 4). Root content of CK(ZR+R) was strongly affected by N types, with higher values as NO_3_^−^ was applied except in *C. chinensis* where the order was reversed (Figure 4). 

### 3.3. Relationships between RA Variables and Root Hormones

Root hormone contents were strongly related to RA variables. When a criterion of *p* < 0.05 was used to retain hormone variables in multiple regression models, we found that IAA showed significant positive correlations with root mass and 1st ORL in these four species, including number of seminal roots and SR length for *O. sativa* in both NO_3_^−^ and NH_4_^+^ treatments. However, we found a negative correlation between IAA content and 1st LRD in *C. chinensis* (Table 4). CK(Z+ZR) and ABA showed significant correlation with RA variables in these four species (Table 4). For ABA and CK(Z+ZR), no consistent patterns were shown across the species, N types and RA variables. Abscisic acid (ABA) was strongly related to root responses as a negative factor three times and positive three times scattered across the species and RA variables (Table 4). Cytokinins (CK(Z+ZR)) were a positive factor two times and negative three times in these species (Table 4).

## 4. Discussion

Using a hydroponic experimental system and four plant species, we identified correlations between nutrient environment, root growth, and hormones that may account for interspecific variation of root responses to nutrient heterogeneity. N type and concentration had strong apparent influences on root growth, while temporal variation in N concentration much less so. Hormone responses were complex and suggest that plant species may use different physiological pathways to create similar root system architectures.

### 4.1. Effects of Nitrogen Treatment on Root Growth and Architecture

Our experiment produced substantial intra- and inter-specific variations in root biomass. In general, NO_3_^−^ resulted in more root growth than NH_4_^+^, but the high concentration of both NO_3_^−^ and NH_4_^+^ reduced root growth when compared to the medium and low concentrations. These results were consistent, at least in part, with other reports. Root growth is often inhibited by high concentrations of NH_4_^+^ [55]. In this study, we observed higher root biomass in the NO_3_^−^ treatments than in the NH_4_^+^ treatments probably due to ammonium toxicity. Studies have reported that plants with NH_4_^+^ as a sole N source can lead to toxicity, especially at high concentrations [56,57]. The negative effect of abundant NH_4_^+^ on plant growth may be caused by induction of nutrient deficiency of other ions or alterations in internal pH [55,56]. 

The temporal variation of nutrient addition had small impacts on root mass growth; however, there were interesting differences across the plant species. Specifically, the variable and stable N treatments induced similar root growth in *A. altissima* and *O. sativa*, however, in *S. canadensis,* root growth was enhanced at the variable low/high nitrate and variable medium/high ammonium treatments. In contrast, total root mass was significantly decreased in *C. chinensis* when nitrogen concentrations were temporally variable (Figure 2). In previous studies, we observed the same negative effects of temporally variable nutrient supply on root growth in *C. chinensis* [58,59].

Two architectural traits of the root systems (1st order root length and density of 1st order laterals) were very responsive to the nitrogen treatments (Figure 3). They tend to be negatively correlated; i.e., fine roots that are sparsely attached along a root axis tend to be longer, while those that are densely packed along a root axis are shorter. In our study, Pearson correlations between the two traits in *C. chinensis*, *S. canadensis*, and *A. altissima* were −0.61, −0.166, and −0.64, with *p*-values of <0.0001, 0.357, and <0.0001, respectively. It is difficult to generalize these results across plant species because most studies examining the impact of external N on the root branching have focused on molecular mechanisms in *Arabidopsis* and a few other model species [16,42]. However, in these studies of model plant species, the negative effects of high NO_3_^−^ or NH_4_^+^ concentrations on lateral root development or elongation have been frequently reported [18,42,57,60]. The seminal and crown roots of maize were inhibited at high NO_3_^−^ concentration of 5 mM [18]. *Arabidopsis* seedlings were grown at 1  mM and 50  mM KNO_3_, and the results showed that the LR development was strongly depressed at the higher NO_3_^−^ concentration [60]. In this study, we found complicated root architecture responses to high concentrations of NO_3_^−^ and NH_4_^+^. The highest (18 mM) concentrations of NO_3_^−^ or NH_4_^+^ led to the least seminal root length in *O. sativa* and the least length of 1st order roots on the other three species (Figure 3). However, the density of 1st order roots was greatest at 18 mM NO_3_^−^ in *C. chinensis* and greatest at 18 mM NH_4_^+^ in *A. altissima* (Figure 3). The different responses may reflect variation among species in the way their roots have adapted to N heterogeneity.

### 4.2. N Treatment Influences on Hormones

Although N type had the greatest overall impact on hormone concentrations (Figure 4), there were strong interactions between N type and N concentration, and these interactions varied across species, adding interesting complexity to existing studies of a more limited set of model plant species [42,61]. In general, however, our study reflects a pattern of lower IAA production when nitrogen is more abundant, which has been found in *Arabidopsis* and Maize [18,61]. This general finding correlates with the proposed model that lower soil nitrogen concentrations result in higher IAA in root tips, leading to a stimulation of root growth and greater capacity to forage for nitrogen. 

### 4.3. Relating Root Hormones to RA Features and Root Mass

We have examined nitrogen treatment impacts on RA and on hormone responses separately. What about the relationships between the two sets of response variables: RA and hormones? When we explored these via multiple regression (Table 4) and by examining the results plotted in Figure 3 and Figure 4, we found that plant species may not share common systems in regulating their root hormone levels and therefore regulating RA responses. While IAA was strongly and positively linked to many RA responses, especially root growth, the root growth of *S. canadensis* in the NO_3_^−^ treatments was positively related to just CK(Z+ZR), which is believed to antagonistically interact with IAA in roots [34]. Abscisic acid (ABA) was positively related with fine root growth for *A. altissima* in the NO_3_^−^ treatments, but negatively related to it for *O. sativa* in NH_4_^+^ treatments (Table 3). Furthermore, the high variability of RA features in *A. altissima* and *O. sativa* would be hard to attribute to these three hormone responses since the latter varied so little (Figure 3 and Figure 4). It is possible that the latter two species responded to the nutrient heterogeneity in our study by altering constituent and induced transporters more than by changing root architecture. Low nitrogen concentrations in aerial parts of *Arabidopsis* can induce production of nitrogen transporters through the expression of the *AtNRT2* gene family [62].

Abscisic acid (ABA) is involved in LR formation, but its role in regulating root elongation and the formation of laterals in repose to N concentrations is not yet completely understood [44]. From studies using an ABA signaling mutant of *Arabidopsis*, it appears that ABA is involved in lateral root formation in complex ways [25,45]. During LR formation, ABA promotes LR initiation and inhibits LR emergence [25]. The inhibitory effect of the high NO_3_^−^ treatment on LR formation in *Arabidopsis* is significantly reduced in ABA insensitive mutants, indicating the involvement of ABA in nitrate-mediated LR formation [38]. The variation among species in ABA response in this study, and the interactions among treatments seen in some species but not in others, suggest complexity in how ABA is influenced by external N conditions and how it affects root growth. 

Because the three root hormones that we studied responded to the same sets of N treatments in species-specific ways, we propose that biochemical pathways underpinning root foraging behavior vary substantially across species. Hormone receptor sensitivity or density may account for some of the observed variation. It is well known that hormone receptors play vital roles by transmitting a hormonal signal into a cellular signaling cascade or by promoting changes in gene transcription [63]. For example, the auxin receptor AFB3 can coordinate primary and lateral root growth by two independent pathways in response to nitrate [64]. Furthermore, it has been reported that different hormone receptors can enhance expression of genes involved in hormone metabolism. Our study did not focus on specific molecular pathways that led to changes in root RA when nutrient availability was varied. 

### 4.4. Limitations

It is possible that the methods we used to assess hormones, due to the sample sizes and other limitations, may not be detailed enough to reveal more consistent underlying patterns than we see in Figure 4 and Table 4. First, we measured the hormone levels at the whole fine root scale; it is possible that this scale of observation failed to detect key within-root points (e.g., within meristems, or root tips), where small changes in hormone concentrations may exert major influences on whole roots that will ultimately affect architecture changes at the root system level. Second, because we analyzed our data in regression using hormone contents as independent values, we may have missed important influences of hormone balances relative to each other. Studies of *Arabidopsis* have shown that cytokinin plays an antagonistic role to auxin in lateral root initiation [25,44]. It has been reported that CK blocked lateral root initiation, but auxin nonetheless played an important positive role in lateral root initiation [65]. Third, there may be other plant hormones, such as brassinosteroids (BR), ethylene, gibberellin (GA) [25], and interactions of hormones that regulate individual root growth, development, and root system behavior [42]. Additionally, as we did not employ an internal standard to measure hormone loss due to our purification protocols, our direct measurements can only be compared to similar proceeded experiments. Finally, we acknowledge that a homogenous hydroponic nutrient environment is not representative of a natural soil system. However, some technical challenges would be faced in performing the same N treatments in soil: (1) it takes an extensive amount of time to clean the roots, which would greatly reduce the accuracy in hormone analyses; (2) the entire root architecture could not be maintained during root excavation and cleaning; and, (3) the samples could not be sampled and scanned simultaneously, leading to potential changes in root hormone content. However, in our hydroponic experiment, these problems were avoided. We acknowledged this trade-off in order to see general patterns and avoid serious technical obstacles. The results are mainly in accordance with those in previous studies. 

## Figures and Tables

**Table 1:** A list of ten nitrogen treatments used in this study: five each for nitrate and ammonium; A and B were variable concentration treatments, while C, D, E were stable concentration treatments.

N Type	N Treatment	N Concentration (mM)	N Applications	Treatment Time Per Period (h)
NO_3_^−^	A	0.2/18	Variable	62/10
	B	1/18	Variable	62/10
	C	0.2	Stable	72
	D	1	Stable	72
	E	18	Stable	72
NH_4_^+^	A	0.2/18	Variable	62/10
	B	1/18	Variable	62/10
	C	0.2	Stable	72
	D	1	Stable	72
	E	18	Stable	72

**Table 2:** Hoagland nutrient solution formulas used in this experiment.

Nutrient Element	Chemical Formula	Concentration (mM)
Ferric salt	FeSO_4_·7H_2_O	0.1
	EDTA·2Na	0.1
Microelement	H_3_BO_4_	0.04
	MnSO_4_·H_2_O	0.002
	ZnSO_4_·7H_2_O	0.001
	CuSO_4_·5H_2_O	0.0003
	KI	0.005
	Na_2_Mo_4_·2H_2_O	0.0001
	CoCl_2_·6H_2_O	0.0002
Macroelement	KH_2_PO_4_	1.2
	MgSO_4_·7H_2_O	1.5
	CaCl_2_·2H_2_O	2

**Table 3:** Multivariate analysis of variance (MANOVA) results illustrating the influence of nitrogen types (NO_3_^−^ and NH_4_^+^), three nitrogen concentrations (0.2 mM, 1 mM and 18 mM) and two ways of nitrogen application (stable and variable) on root mass, contents of IAA, ABA and CK(Z+ZR) in roots, and root architecture (1st order roots length (1st ORL), seminal root length (SR length) for *O. sativa*, inter-length of 1st OR (IBLLR), density of 1st OR (1st LRD) and seminal root number (SR#) for *O. sativa*).

Source	df	FR Mass (g)		1st ORL (mm)		IBLLR		1st LRD		df	IAA (ng/g)		ABA (ng/g)		CK(ZR+R) (ng/g)	
		MS	F/sig.	MS	F/sig.	MS	F/sig.	MS	F/sig.		MS	F/sig.	MS	F/sig.	MS	F/sig.
*Callistephus chinensis*																
Model correction	9	0.0035	38.10 ***	45,354.5	74.32 ***	144.06	3.12 **	0.0013	21.90 ***	9	206,037	104.8 ***	12,879	15.5 ***	5781.5	30.5 ***
N-type (A)	1	0.011	105.73 ***	108,514	177.8 ***	26.19	0.57	0.0099	162.1 ***	1	1,584,583	806.3 ***	67,765	81.5 ***	49,216	260 ***
Appl. trt. (B)	1	0.008	78.12 ***	79,321.4	78.12 ***	5.89	0.13	7.69 × 10^−6^	0.54	1	63,582.8	32.35 ***	5548.3	6.67 *	71.4	0.38
Conc. (C)	2	0.0085	85.38 ***	98,897.4	162.1 ***	94.82	2.05	2.73 × 10^−4^	4.49 *	2	83,319.9	42.4 ***	10,477	12.6 ***	15.90	0.084
AB	1	0.0005	4.85 *	391.55	0.642	282.02	6.10 *	7.65 × 10^−5^	1.26	1	17,541.8	8.93 **	6419	7.72 **	20.57	0.11
AC	2	0.0005	4.853 *	16,213	26.57 ***	385.70	8.35 **	6.88 × 10^−4^	11.31 ***	2	3150.7	1.60	10,485	12.6 ***	151.40	0.80
BC	1	0.0003	3.11	5792.3	9.491 **	85.29	1.85	1.17 × 10^−5^	0.19	1	2890.8	1.47	4138.6	4.98 *	141.40	0.75
ABC	1	0.0004	4.252 *	750.4	1.23	21.12	0.46	1.23 × 10^−6^	0.02	1	5204.7	2.65	5303.7	6.38 *	457.50	2.42
Error	32	0.00001		610.278		46.22		6.09 × 10^−5^		31	1965.3		831.4		189.30	
Total	41									40						
*Solidago canadensis*																
Model correction	9	0.081	13.54 ***	14,263.3	9.62 ***	362.4	7.94 ***	0.0002	6.50 ***	9	86,667.4	55.81 ***	34,484	35.1 ***	2336.3	16.5 ***
N-type (A)	1	0.185	31.02 ***	4563.56	27.71 ***	1155.3	25.33	0.001	17.2 ***	1	746,394	480.6 ***	969.8	0.99	14,487.2	102.1 ***
Appl. trt. (B)	1	0.04	0.67	1944.77	11.81 **	305.4	6.69 *	0.0003	9.00 **	1	98.34	0.063	780.5	0.80	1601	11.3 **
Conc. (C)	2	0.063	10.63 **	2759.3	16.75 ***	745.56	16.3 ***	0.0005	13.5 ***	2	5557	3.58 *	4853.5	4.94 *	1061.6	7.48 **
AB	1	0.044	7.34 *	5.25	0.032	146.1	3.2	0.00005	1.47	1	2100	8.93 **	15.857	0.016	1310.3	9.24 **
AC	2	0.077	12.93 ***	64.88	0.39	55.43	1.22	0.00004	1.13	2	4757.8	3.06	6273.9	6.39 **	788.9	5.56 *
BC	1	0.011	1.84	2441.6	14.82 **	18.59	0.41	0.00001	0.14	1	12,482	8.04 *	142,701	145 ***	314.84	2.22
ABC	1	0.17	28.44 ***	1356.6	8.24 *	110.8	2.43	0.00003	0.66	1	5454.79	3.51	131,337	134 ***	324	2.21
Error	18	0.006		164.71		45.62		0.000035		22	1552.91		981.85		141.86	
Total	28									31						
*Ailanthus altissima*																
Model correction	9	0.00085	12.56 ***	7849.1	42.41 ***	431.33	4.35 **	0.0002	4.96 *	9	65,961.4	35.72 ***	415.6	3.70 **	3665.7	100.7 ***
N-type (A)	1	0.0034	50.10 ***	8408	45.45 ***	399.8	4.03	0.00013	453 ***	1	471,249	255.2 ***	3103.9	27.7 ***	30,343.5	833.3 ***
Appl. trt. (B)	1	0.00001	0.17	173.84	0.94	103.2	1.04	0.00001	3.22	1	121.08	0.066	12.29	0.11	70.83	1.95
Conc. (C)	2	0.001	14.78 ***	9233.5	49.9 ***	548.4	5.53 *	0.00039	14.7 ***	2	13,051.3	7.07 **	116.1	1.04	466.5	12.81 ***
AB	1	0.00002	0.30	37.27	0.20	35.618	0.36	0.00004	1.60	1	520.26	0.28	47.11	0.42	2.423	0.067
AC	2	0.00065	9.53 **	4748.4	25.7 ***	436.8	4.40 *	0.00014	5.28 *	2	8631.3	4.67 *	61.76	0.55	78.99	2.17
BC	1	0.00018	2.62	12,057	65.18 ***	442.97	4.46 *	0.00025	9.43 **	1	3409.6	1.85	20.67	0.18	57.11	1.57
ABC	1	0.00013	1.87 *	10,548	57.02 ***	1016.8	10.30 **	0.00036	13.29 **	1	280.19	0.15	16.65	0.15	2.86	0.078
Error	25	0.000007		184.99		99.22		0.00003		21	1846.71		112.22		36.41	
Total	34									30						
*Oryza sativa*																
Model correction	9	0.008	22.45 ***	160,207	40.70 ***	0.36	0.46	188.97	6.06 ***	9	146,533	347.3 ***	987.4	8.81 ***	1845.34	95.3 ***
N-type (A)	1	0.025	70.84 ***	108,655	27.60 ***	1.02	1.30	103.56	3.32	1	1,087,922	2579 ***	5678.7	50.7 ***	15,212	786 ***
Appl. trt. (B)	1	1.08 × 10^−5^	0.031	12,518.9	3.18	0.23	0.29	98.78	3.17	1	1979.9	4.69 *	5.438	0.049	41.19	2.22
Conc. (C)	2	0.016	45.73 ***	467,408	118.74 ***	0.36	0.46	438.24	14.0 ***	2	11,209.9	26.6 ***	33.89	0.30	198.4	1.75
AB	1	3.59 × 10^−5^	0.10	1329.99	0.34	0.18	0.23	138.13	4.43 *	1	3543.1	8.38 **	11.66	0.10	33.87	1.75
AC	2	0.001	1.78	53,198.5	13.52 ***	0.095	0.12	8.89	0.29	2	2389.5	5.66 *	376.3	3.36 *	201.36	10.4 **
BC	1	0.002	4.84 *	2.62	0.001	0.56	0.71	17.71	0.57	1	1784.9	4.23 *	10.05	0.09	56.68	2.93
ABC	1	0.001	3.03	16,682.1	4.24 *	0.53	0.67	5.73	0.18	1	1785.08	4.23 *	2.03	0.018	45	2.33
Error	32	0.00		3936.27		0.79		31.2		23	421.881		112.03		19.35	
Total	41									32						

All the original data were examined heteroscedasticity in Levene’s test, and were log transferred, tested homoscedasticity before MANOVA were processed. In the table: * refers to *p* ≤ 0.05, ** *p* ≤ 0.001, and *** *p* ≤ 0.0001, A refers to nitrogen types (NO_3_^−^ and NH_4_^+^), B refers to two ways of nitrogen application (stable vs. variable), C refers to nitrogen concentrations (0.2 mM, 1.0 mM, 18.0 mM), MS refers to mean squares, F refers to F value, sig. refers to significance, df refers to degrees of freedom, FR refers to fine root.

**Table 4:** Regressions relationships between fine root mass and hormones, RA and hormones. Factors tested with *p*-value >0.05 are excluded.

Species	N Types	Dependent Variable	Regression	Significance	*R*^2^
*Callistephus chinesis*	NO_3_^−^	Root Mass (g)	=−0.013 + 0.78 (IAA)	0.00	0.61
		1st ORL (mm)	=0.036 + 0.49 (IAA)	0.021	0.33
		1st LRD (#/mm)	=−0.041 − 0.54 (ABA)	0.009	0.50
	NH_4_^+^	Root Mass (g)	=0.035 + 0.96 (IAA)	0.00	0.90
		1st ORL (mm)	=0.063 + 0.89 (IAA)	0.00	0.82
		1st LRD (#/mm)	=0.016 − 0.74 (IAA)	0.024	0.28
*Solidago canadensis*	NO_3_^−^	Root Mass (g)	=−0.10 + 0.82 (CK(ZR+R))	0.018	0.47
		1st ORL (mm)			
		1st LRD (#/mm)	=−0.14 + 0.90 (CK(ZR+R))	0.002	0.71
	NH_4_^+^	Root Mass (g)	=0.22 + 0.71 (IAA)	0.012	0.51
		1st ORL (mm)	=0.23 + 0.71 (IAA) − 0.45 (CK(ZR+R))	0.004	0.60
		1st LRD (#/mm)			
*Ailanthus altissima*	NO_3_^−^	Root Mass (g)	=0.28 + 0.68 (IAA) + 0.42 (ABA)	0.01	0.44
		1st ORL (mm)	=0.031 + 0.44 (IAA) − 0.40 (CK(ZR+R))	0.009	0.54
		1st LRD (#/mm)			
	NH_4_^+^	Root Mass (g)	=−0.23 + 0.56 (IAA)	0.050	0.40
		1st ORL (mm)	=−0.39 − 0.94 (ABA)	0.015	0.60
		1st LRD (#/mm)			
*Oryza sativa*	NO_3_^−^	Root Mass (g)	=−0.097 + 0.97 (IAA)	0.049	0.39
		#Seminal Roots (SR)	=−0.14 + 0.50 (IAA) + 0.26 (ABA) − 0.42 (CK(ZR+R))	0.00	0.92
		Length of SR (mm)	=−0.19 + 0.88 (IAA) + 0.28 (ABA)	0.00	0.91
		IBLLR			
	NH_4_^+^	Root Mass (g)	=−0.082 + 0.37 (IAA) − 0.36 (ABA)	0.005	0.48
		#Seminal Roots (SR)			
		Length of SR (mm)			
		IBLLR			
